# Clonal Evolution and Blast Crisis Correlate with Enhanced Proteolytic Activity of Separase in *BCR-ABL* b3a2 Fusion Type CML under Imatinib Therapy

**DOI:** 10.1371/journal.pone.0129648

**Published:** 2015-06-18

**Authors:** Wiltrud Haaß, Helga Kleiner, Christel Weiß, Claudia Haferlach, Brigitte Schlegelberger, Martin C. Müller, Rüdiger Hehlmann, Wolf-Karsten Hofmann, Alice Fabarius, Wolfgang Seifarth, Schweizerische Arbeitsgemeinschaft für Klinische Krebsforschung (SAKK) and the German CML Study Group

**Affiliations:** 1 III. Medizinische Universitätsklinik (Hämatologie und Onkologie), Medizinische Fakultät Mannheim der Universität Heidelberg, Mannheim, Germany; 2 Abteilung Medizinische Statistik und Biomathematik, Medizinische Fakultät Mannheim der Universität Heidelberg, Mannheim, Germany; 3 MLL Münchner Leukämielabor, München, Germany; 4 Institut für Zell- und Molekularpathologie, Medizinische Hochschule, Hannover, Germany; Uppsala University, SWEDEN

## Abstract

Unbalanced (major route) additional cytogenetic aberrations (ACA) at diagnosis of chronic myeloid leukemia (CML) indicate an increased risk of progression and shorter survival. Moreover, newly arising ACA under imatinib treatment and clonal evolution are considered features of acceleration and define failure of therapy according to the European LeukemiaNet (ELN) recommendations. On the basis of 1151 Philadelphia chromosome positive chronic phase patients of the randomized CML-study IV, we examined the incidence of newly arising ACA under imatinib treatment with regard to the p210BCR-ABL breakpoint variants b2a2 and b3a2. We found a preferential acquisition of unbalanced ACA in patients with b3a2 vs. b2a2 fusion type (ratio: 6.3 vs. 1.6, p = 0.0246) concurring with a faster progress to blast crisis for b3a2 patients (p = 0.0124). *ESPL1*/Separase, a cysteine endopeptidase, is a key player in chromosomal segregation during mitosis. Separase overexpression and/or hyperactivity has been reported from a wide range of cancers and cause defective mitotic spindles, chromosome missegregation and aneuploidy. We investigated the influence of p210BCR-ABL breakpoint variants and imatinib treatment on expression and proteolytic activity of Separase as measured with a specific fluorogenic assay on CML cell lines (b2a2: KCL-22, BV-173; b3a2: K562, LAMA-84). Despite a drop in Separase protein levels an up to 5.4-fold increase of Separase activity under imatinib treatment was observed exclusively in b3a2 but not in b2a2 cell lines. Mimicking the influence of imatinib on BV-173 and LAMA-84 cells by *ESPL1* silencing stimulated Separase proteolytic activity in both b3a2 and b2a2 cell lines. Our data suggest the existence of a fusion type-related feedback mechanism that posttranslationally stimulates Separase proteolytic activity after therapy-induced decreases in Separase protein levels. This could render b3a2 CML cells more prone to aneuploidy and clonal evolution than b2a2 progenitors and may therefore explain the cytogenetic results of CML patients.

## Introduction

The BCR-ABL tyrosine kinase (TK) formed by the balanced translocation t(9;22)(q34;q11) is the “key player” in the pathogenesis of chronic myeloid leukemia (CML). Its deregulated TK activity affects various downstream signaling pathways and results in reprogramming of the prior lineage commitment of hematopoietic stem and early progenitor cells. [1] Compromising multiple aspects of the affected hematopoietic stem cell, including proliferation, apoptosis, cell to cell signaling and differentiation, the BCR-ABL oncoprotein triggers aberrant clonal hematopoiesis and drives disease progression from chronic phase (CP) toward the fully transformed phenotype of blast crisis (BC). [2]

Imatinib (IM) is a selective TK inhibitor (TKI) and represents one of the current first line treatment options for CML. [3] However, persistence of so-called leukemia stem cells (LSCs) with low BCR-ABL expression, insensitivity to IM treatment and long term survival capacity has been observed. [4] Acquisition of additional genetic lesions in LSCs or their progeny drives leukemic transformation from CML CP to accelerated phase (AP) or BC. [5]

Genomic instability and aneuploidy are hallmarks of the progressing CML and concur with BCR-ABL mutations encoding resistance to TKI and/or development of additional chromosomal aberrations (ACA) in addition to the Philadelphia chromosome (Ph) (= clonal evolution). [6,7] About 35% of patients in CP develop resistance or intolerance to IM and frequently undergo clonal evolution. [8,9] While approximately 10–12% of patients in CML CP display ACA at diagnosis, this proportion of patients rises to approximately 30% and 80% in AP and BC, respectively. [10,11] Recently, we have shown that major route ACA (all unbalanced, e.g. second Ph, trisomy 8, isochromosome 17q, or trisomy 19) at diagnosis are associated with a negative impact on survival and signify progression to AP and BC. [12] Moreover, clonal evolution during the course of CML is considered a feature of acceleration and indicate poor prognosis as patients with ACA show lower cytogenetic response rates under IM. [6,13,14] According to the European LeukemiaNet (ELN) recommendations newly arising ACA under IM treatment define failure of therapy. [3,15]

The occurrence of supernumerary centrosomes (= centrosome amplification) is the major cause of multipolar mitotic spindle formation and chromosomal missegregation leading to chromosomal instability (CIN) and aneuploidy. [16–18] Centrosome amplification, in particular, the accumulation of additional centrosomes (n>2), is frequently detected in solid and hematological human cancers and has already been found in pre-neoplastic lesions i.e. early stages of carcinogenesis. [19,20] CIN is considered to continually drive clonal evolution and tumor heterogeneity. [21,22]

In CML, centrosome amplification is an early event in the transformation process and occurs at the earliest identifiable step in CML development. [17] Recently, in a long-term *in vitro* study on a CML CP model we have established the functional link of p210BCR-ABL TK activity with centrosome amplification and clonal evolution. [23] This is in accordance with the observation that p210BCR-ABL and c-ABL are both centrosome associated proteins. [24] However, IM treatment did not prevent the development of centrosome amplification; but by itself induced centrosomal and/or cytogenetic alterations in several *bcr-abl*-negative cell line models and *in vivo*. [25–29]

The maintenance of regular centrosome numbers in non-malignant cells depends on the ordered centriole duplication strictly limited to once per cell cycle. Disengagement of mother and daughter centriole is a prerequisite for semiconservative centriole duplication and is provided by proteolytic cleavage of cohesin, a “glue” multi-protein complex that is also responsible for sister-chromatide cohesion. [30–32] Separase, a cysteine endopeptidase encoded by the gene *espl1*, conducts cleavage of cohesin. [32–34] Ectopic activation of Separase proteolytic activity causes premature sister-chromatide separation and centriole disengagement and overexpression of Separase has been reported to induce centrosome amplification, aneuploidy and tumorigenesis. [31,35] Recently, striking functional evidence for the oncogenic activity of deregulated Separase was provided by Pati and coworkers who have generated a transgenic *MMTV-Espl1* mouse model that overexpresses Separase protein in the mammary gland. These mice developed aggressive and highly aneuploid mammary carcinomas with high levels of CIN and cell cycle defects including multiple centrosomes and multinucleated cells. [36] Moreover, Separase overexpression has been considered as potential predictor of progression-free survival and relapse in glioblastoma. [37]

The proteolytic activity of Separase is tightly regulated by multiple inhibitory mechanisms combining Securin binding, specific serine residue phosphorylation (pSer1126) by CyclinB1/Cdk1, autocatalytic cleavage and PP2A-dependent stabilization of Separase-bound Securin. [38–41] The finding that *espl1*/Separase acts as an oncogene/-protein in various cancers including CML renders this protease a key target to unravel the molecular mechanisms involved in the development of centrosome amplification and clonal evolution in IM-treated CML. [24,27,35,42]

In this study, we used the cytogenetic data from CML study IV to investigate incidences of newly arising unbalanced ACA and blast crises under IM long-term treatment with regard to the p210BCR-ABL breakpoint variants b2a2 and b3a2. In search for potential underlying molecular mechanisms we performed protein biochemical studies on Separase expression and activity profiles in a panel of b2a2 and b3a2 CML cell lines. Our data point to the existence of a p210BCR-ABL-dependent feedback mechanism that posttranslationally stimulates Separase proteolytic activity after an IM-induced decrease in Separase protein levels exclusively in b3a2 cells. As a known promoter of aneuploidy and clonal evolution hyperactive Separase is an excellent candidate for explaining the cytogenetic *in vivo* data.

## Materials and Methods

### Cell lines and culture conditions

Six human cell lines were investigated. NHDF was derived from Promocell GmbH (Heidelberg, Germany). KCL-22, BV-173, LAMA-84 and K562 were obtained from the DSMZ (German Collection of Microorganisms and Cell Cultures, Braunschweig, Germany). The immortalized human urothelial cell line UROtsa was a gift of the Department of Urology, Mannheim Medical Center, Mannheim Germany and cultured as previously described. [43] All cells were cultured in RPMI-1640 supplemented with 10% fetal bovine serum, 2% glutamine and 1% penicillin-streptomycin (Gibco/Invitrogen, Karlsruhe, Germany) at 37°C in 5% CO_2_ atmosphere. Exponentially growing cells were used in at least triplicate experiments.

### Patients and ethics statement

Clinical and cytogenetic data of 1151 patients with Ph+ and BCR-ABL+ CP CML randomized to the German CML-Study IV (imatinib 400 mg vs. imatinib 800 mg vs. imatinib in combination with IFN vs. imatinib in combination with low-dose cytarabine vs. imatinib after IFN failure) were investigated. [12] Mean observation time was 5.6 years. There were 459 female (40%) and 692 male (60%) patients with a median age of 53 years (range, 16–88); median age was lower in ACA patients (48 years). The definitions of CML phases followed the ELN recommendations. [3] The protocol followed the declaration of Helsinki and was approved by the *IRB/Medizinische Ethikkommision II der Medizinischen Fakultät Mannheim der Ruprecht-Karls-Universität Heidelberg*. (http://www.umm.uni-heidelberg.de/inst/ethikkommission, # 0214.6 from 2002-08-21). Written informed consent was obtained from all patients before they entered the study. The CML study IV is registered at www.clinicaltrials.gov as # NCT00055874.

### Imatinib treatment

Cells were treated with IM (Biomol GmbH, Hamburg, Germany) in concentrations of 1 to 10μM for 24h (KCL-22, LAMA-84, K562), 48h (BV-173) and 6d (NHDF, UROtsa). Untreated cells served as controls.

### Separase silencing by espl1-directed siRNA

*Espl1*-specific siRNA (FlexiTube GeneSolution GS9700 for *espl1*) was purchased from Qiagen, (Hilden, Germany). As negative controls the same cells were transfected with AllStars Negative Control siRNA (Qiagen) a nonsilencing siRNA with no homology to any known mammalian gene. Transfection was accomplished using the Nucleofector manual (program T016, Lonza GmbH, Cologne, Germany). For siRNA treatment, 5x10^6^ BV-173 cells and 2x10^6^ LAMA-84 cells were resuspended in 100 μl Cell Line Nucleofector Solution V (LAMA-84) or Solution R (BV-173) (Lonza) containing 18 μl Supplement S Solution (Lonza). SiRNA was added to a final concentration of 0.01 nmol per 10^6^ cells.

### Quantification of espl1 transcripts by qRT-PCR

Total RNA was extracted using RNeasy kit (Qiagen) and reverse transcribed using Superscript II kit (Gibco/Invitrogen). For quantification of separase (*espl1*) transcript levels, the commercial Hs_ESPL1_1_SG QuantiTect Primer Assay (Qiagen) was employed according to the instructions (two-step LightCycler 480 protocol) of the manufacturer. For normalization, the housekeeping gene beta-glucuronidase (*gus*, NM_000181, GUSB, primer set Hs_GUSB_1_SG, QuantiTect Primer Assay, Qiagen) was amplified. QRT-PCR was performed with the Roche LightCycler 480 System, using LC480 DNA Master SYBR Green and the standard LightCycler protocol (Roche Diagnostics, Mannheim, Germany). Relative transcript levels calculated from triplicate measurements were calculated by the 2^-∆∆CT^ method with values normalized to *gus* and relative to transcription in untreated control cells. [44]

### Western blot analysis, antibodies

Western blot immunostaining of p210BCR-ABL, pCrkL, Separase, Securin, CyclinB1 and Actin was performed as described previously. [42]

### Karyotype analysis

Cytogenetic analysis was performed as described previously. [26] At least 20 metaphases were analyzed by G- or R-banding technique and interpreted according to the International System for Human Cytogenetic Nomenclature (ISCN 2009).

### Separase activity assay

About 60 μg cleared native protein lysate was analyzed in a quantitative fluorogenic assay according to Basu and coworkers. [45] Spectrofluorometry was performed as described previously. [42]

### Statistical analysis

All statistical calculations have been done with GraphPad Prism software version 5.0 (GraphPad Inc., La Jolla, USA) or SAS software, release 9.3 (SAS Institute Inc.,Cary, NC, USA). Quantitative parameters are presented as mean values together with standard deviation (SD) or 95% confidence intervals (CI). For qualitative data, absolute and relative frequencies are given. In order to compare two mean values, two-tailed unpaired t tests have been used. Frequencies have been compared using Chi^2^ or Mann-Whitney-U-tests. All tests have been performed 2-sided. Test results with p<0.05 were considered significant. Values between p≥0.05 and p≤0.1 were defined as trend.

## Results

### Long-term survey of IM-treated CML patients confirms preferential acquisition of unbalanced ACA in patients with the b3a2 fusion type of p210BCR-ABL

To investigate the conditional context between *bcr-abl* breakpoint variant (b3a2 or b2a2), IM therapy and acquisition of ACA and clonal evolution we have analyzed therapy-related acquisition of ACA in 1151 patients under IM treatment that were randomized to the CML-Study IV. [12] To test for diverse ACA acquisition rates in b2a2 vs. b3a2 CML patients, all patients (n = 1151) were classified according to their *bcr-abl* breakpoint variant and the appearance of ACA during the course of the disease that were not detectable at diagnosis (before IM treatment). As given in Table 1, a total of 978 patients (463 with b2a2 and 515 with the b3a2 variant) were selected from this study. 173 of 1151 patients (15%) were omitted because they carried both or switched (n = 7) between b2a2 and b3a2 *bcr-abl* breakpoint variants within the observation period. Of the remaining patients (n = 978) ACA acquisitions during IM therapy were discovered in 36 (7.8%) and 29 (5.6%) patients with the b2a2 and b3a2 variant, respectively.

**Table 1:** Occurrence of ACA in CML patients (n = 978) of the randomized CML Study IV during long term treatment with IM.

Numbers and patient characteristics	b2a2 (e13a2)	b3a2 (e14a2)
t(9 ;22)(q34 ;q11),—ACA	427 (92.2%)	486 (94.4%)
t(9 ;22)(q34 ;q11), + ACA	36 (7.8%)	29 (5.6%), p = 0.1789
	463 (100%)	515 (100%)

ACA, additional clonal cytogenetic alteration

Since chromosomal missegregations are the first line consequences of defective mitotic spindles concurring with supernumerous centrosomes (associated with overactive Separase), the observed ACA were further classified with respect to the types “balanced” (no change in DNA content, e.g. reciprocal translocation) or “unbalanced” (chromosomal losses and gains) (Table 2). The latter serves best as a direct measure of mitotic spindle failure as no additional DNA modifying enzymes (e.g. DNA polymerases of DNA repair) are involved. While in the b2a2 group the proportion of type “unbalanced” to “balanced” was 22:14 (61%:39%, ratio 1.6), a ratio of 25:4 (86%:14%, ratio 6.3) was found in the b3a2 patient group (p = 0.0246). The preferential occurrence of unbalanced ACA in the Ph+ clone (for karyotypes see S1 Table) of IM treated patients with the b3a2 fusion type coincides with a faster progress to blast crisis for b3a2 patients (p = 0.0124, median and range in months: 9 (3–15) vs. 25 (6–48). This suggests the existence of a molecular mechanism that favours disease progress via chromosomal missegregation, aneuploidy and clonal evolution specifically in p210BCR-ABL progenitors with the b3a2a fusion type. No further differences between b3a2 and b2a2 patient groups regarding distinct abnormalities in hematologic features or in time under IM until detection of ACA (b2a2: 23 (2–87); b3a2: 21 (1–101) were found.

**Table 2:** Classification of ACA arisen during long term treatment with IM.

Numbers and patient characteristics	b2a2 (e13a2): n = 36	b3a2 (e14a2): n = 29
t(9;22)(q34 ;q11) + ACA unbalanced	22 (61%)	25 (86%)
t(9 ;22)(q34 ;q11) + ACA balanced	14 (39%)	4 (14%)
Ratio (ACA unbalanced/balanced) *	1.6	6.3, p = 0.0246
Blast crises within observation period *	8 (22%)	7 (24%), p = 0.8554
Time to unbalanced ACA, median and range [months] ^#^	23 (2–87)	21 (1–101), p = 0.9150
Time to BC, median and range [months] ^#^	25 (6–48)	9 (3–15), p = 0.0124

ACA, additional clonal cytogenetic alteration

* Chi2-test

^#^ Mann-Whitney-U-test

### Experimental design and cell line characterization

In search for potential underlying molecular mechanisms we performed comparative studies on Separase expression and activity profiles in a panel of b2a2 and b3a2 CML cell lines. The experiments aimed at confirming and expanding previously published work. [42] Here, analogous cell culture experiments were performed on six human cell lines (Table 3) with focus on the comparison between b2a2 (KCL-22, myeloblastic; BV-173, lymphoblastic) and b3a2 cell lines (LAMA-84, myeloblastic; K562, lymphoblastic). Primary NHDF cells and SV-40 immortalized UROtsa served as models for non-malignant cells. Analyzing the changes that occur within the first few rounds of the cell cycle after IM administration, our experimental setting should provide insight into Separase post-translational regulatory mechanisms occuring immediately after IM administration before any phenotypic alterations in centrosomal or cytogenetic status may manifest. Since the proteolytic activity of Separase in non-malignant cells is tightly restricted to the mitotic anaphase and is therefore regulated in a cell cycle-dependent manner, treatment periods were chosen with respect to the respective cell doubling times so that at least one cell cycle round was completed under IM treatment and less than 12% of cells were apoptotic. We considered cell doubling times as the best approximative estimation of cell cycle transits albeit we are aware that these values do not reflect potential loss of cells by cell death and delayed exit of cells from the cell cycle. However, we assigned 6d (NHDF, UROtsa), 48h (BV-173) and 24h (KCL-22, LAMA-84, K562) of treatment as appropriate before cell harvesting and target analysis (Table 3). All cell lines were treated with therapeutic doses of IM (range: 1 to 10 μM) as performed in our previous studies. [23,26,27,42] In accordance with data from extensive studies on the dose-dependent effects and time kinetics of IM we applied lower IM doses (range: 1 μM to 2.5 μM) for leukemia-derived p210BCR-ABL-positive cells (KCL-22, BV-173, K562 and LAMA-84) than for p210BCR-ABL-negative cells (range: 2.5 μM to 10 μM). [46–48] Treating CML cell lines with IM doses higher than 2.5 μM for a longer period than 48h impeded the collection of enough viable cells for Western blot analysis and Separase activity assays (data not shown).

**Table 3:** Origin and characteristics of human cell line models under investigation.

Cell line	Cell type	Origin	Doubling time [h] ^1^	BCR-ABL-copy no/ type	centrosomal status [%] ^2^	Cytogenetics	Used as model for
NHDF	normal human dermal fibroblasts, primary	juvenile foreskin, from healthy donor	~ 72	none	normal, < 5%	46,XY	normal cells
UROtsa	human urothelial	normal urothelial cells immortalized with SV40	~ 63	none	normal, < 3%	46,XX	normal cells
KCL-22	human CML (BC)	pleural effusion of patient with CML BC, myeloblastic	~24	2 / (b2a2)	aberrant, 78%	human hyperdiploid karyotype with 3.3% polyploidy– 51(47–51)<2n>X,-X, +1, +6, +8, +8, +14, +22, del(1)(p22), t(9;22)(q34;q11), add(17)(p12-13), i(21q), der(22)t(9;22)(q34;q11)–carries Phx2 –matches published karyotype (DSMZ)	CML, b2a2
BV-173	human CML (BC)	PB of a patient with CML BC, lymphoblastic	~48	1 / (b2a2)	aberrant, 10%	human hyperdiploid karyotype– 47(46–48)<2n>X/XY, -9, +22, +mar, add(1)(q42), add(8)(p23), t(9;22)(q34;q11), der(22)t(9;22)(q34;q11), der(?)t(9;?)(?p11;?) (DSMZ)	CML, b2a2
LAMA-84	human CML (BC)	PB of a patient with CML BC, myeloblastic	~ 50	4 / (b3a2)	aberrant, 43%	73/74(69–77)<3n>XX,-X,+1,-2,+5,+6,+8,+13,-14,+17,+17,-18,+22,+mar,del(7)(p15),der(9)t(9 ;22)(q34 ;q11)x2,i(11q), add (13)(q33),del(17)(p12),der(22)t(9 ;22)(q34 ;q11)x4 (DSMZ)	CML, b3a2
K562	human CML (BC)	pleural effusion of a patient with CML BC, lymphoblastic	~ 36	11 / (b3a2)	aberrant, 34%	61–68<3n>XX,-X,-3,+7,-13,-18,+3mar,del(9)(p11/13), der(14)t(14;?)(p11;?),der(17)t(17;?)(p11/13;?),der(?18) t(15;?18)(q21;?q12),del(X)(p22) (DSMZ)	CML, b3a2

Abbreviations: PB, peripheral blood; CML, chronic myeloid leukemia; CP, chronic phase; BC, blast crisis; no, number; DSMZ, German Collection of Microorganisms and Cell Cultures, Braunschweig, Germany

^1^ doubling time of subconfluent cells

^2^ Analysis of 100 cells by immunofluorescence microscopy after centrosome (pericentrin) staining

**Fig 1:**
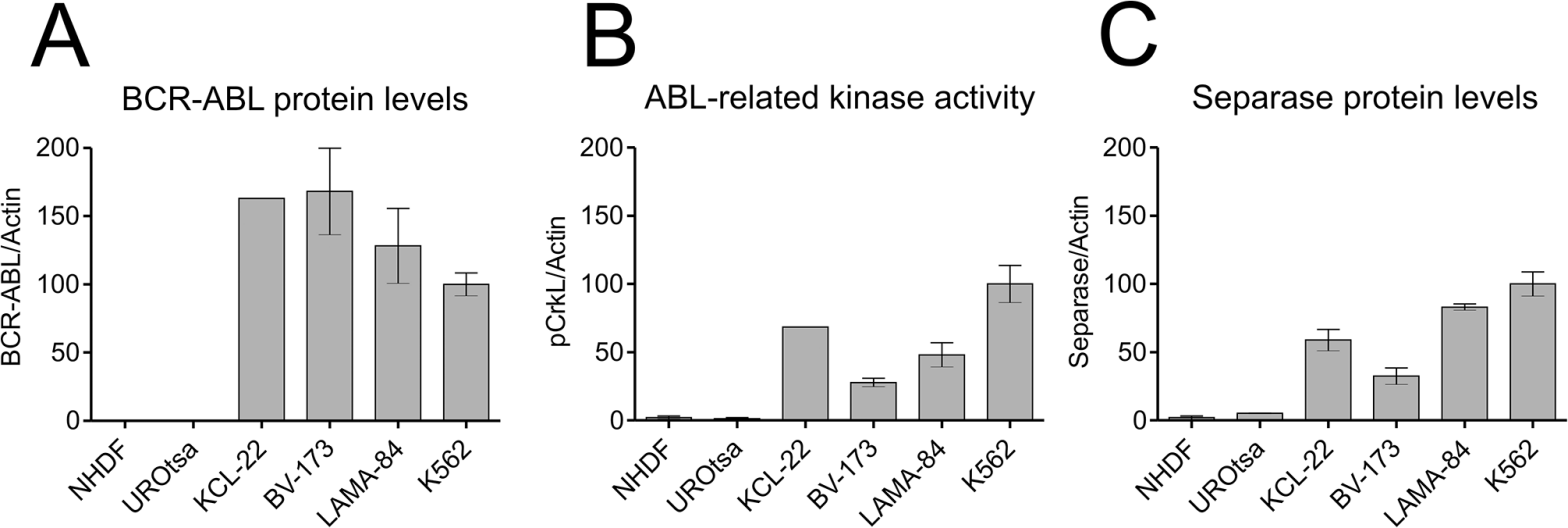
Protein and activity levels of p210BCR-ABL and Separase expression in cell lines under investigation. Protein levels of p210BCR-ABL (**A**), pCrkL (**B**) and Separase (**C**) based on densitometric evaluation of immunostained Western blots were normalized to Actin as loading control. Abl-related TK activity (BCR-ABL + c-ABL) was measured as pCrkL/Actin **(B)**. Analyses were performed on protein lysates derived from p210BCR-ABL-positive (KCL-22, BV-173, LAMA-84, K562) and-negative non-malignant cells (NHDF, UROtsa). KCL-22 and BV-173 carry the *bcr-abl* breakpoint variant b2a2, whereas LAMA-84 and K562 display the b3a2 fusion gene variant. All values refer to that of K562 (= 100%).

All untreated cell lines were tested thoroughly with respect to *bcr-abl* expression, karyotype and centrosome status, and proliferation rate. The protein levels of p210BCR-ABL, phosphorylation of CrkL at Tyr207 (pCrkL) as surrogate marker of ABL- and p210BCR-ABL-related kinase activity and of Separase were analyzed (Fig 1). As expected, p210BCR-ABL protein was detected exclusively in *bcr-abl*-positive cell lines (Fig 1A). The b2a2 variant cell lines KCL-22 and BV-173 displayed higher levels of p210BCR-ABL protein (163.1% and 168.2 +/- 77.6(SD)%, respectively) than the b3a2 CML cell lines LAMA-84 and K562 (128 +/- 61.3(SD)% and 100.0 +/- 18.7(SD)%, respectively). Densitometric analysis of pCrkL (Fig 1B) revealed the highest phosphorylation levels in K562 (= 100% phosphorylation), followed by KCL-22 (68.6%), LAMA-84 (48.2 +/- 20(SD)%) and BV-173 (27.8 +/- 7.6(SD)%) pointing to the constitutive TK activity of p210BCR-ABL. Minor phosphorylation levels for pCrkL were detected in NHDF (2.1 +/- 1.9(SD)%) and UROtsa cells (1.4 +/- 1.3(SD)%).

Separase protein level analysis revealed a general overexpression (range 16 to 50-fold) in all BCR-ABL-positive cells when compared to NHDF cells (Fig 1C). This is in line with various reports on separase overexpression in cancers, including CML. [24,35] Moreover, Separase protein levels correspond to observed doubling times and p210BCR-ABL TK activity, as the fast-growing leukemic cells (KCL-22, BV-173, LAMA-84 and K562) displayed higher Separase protein levels (relative protein levels of 59 +/- 11(SD)%, 33 +/- 15(SD)%, 83 +/- 5.3(SD)% and 100 +/- 20(SD)%, respectively) than *bcr-abl*-negative slow-growing cells (NHDF 72h doubling time, relative protein level 2.0 +/- 3.0(SD)%; UROtsa 63h doubling time, relative protein level 5.3 +/- 0.3(SD)%. Peak pattern comparison of graph B and graph C (Fig 1) suggests that Separase expression correlates positively with p210BCR-ABL TK activity confirming our recent results on U937p210BCR-ABL/c6 cells with inducible p210BCR-ABL expression (Tet-On). [42]

### Separase protein levels decrease and Separase proteolytic activity increases exclusively in b3a2 p210BCR-ABL-positive cell lines under IM treatment

**Fig 2:**
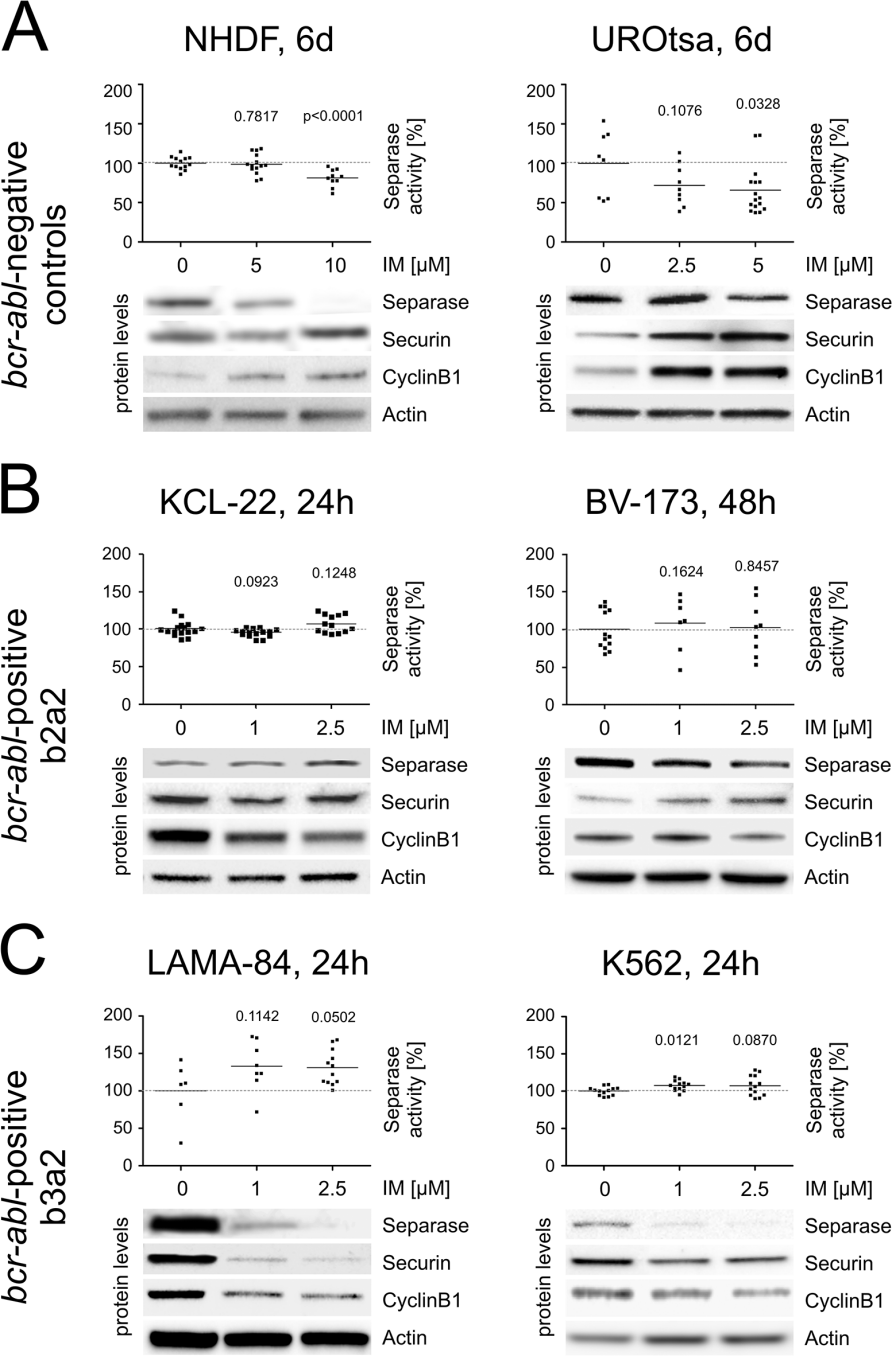
Separase proteolytic activity and levels of master Separase proteolytic activity regulators in *bcr-abl*-negative (A) and–positive cell lines (B, C) treated with IM. Cells were treated with IM for times and doses [μM] given on top. Separase proteolytic activity was quantified using an *in vitro* fluorometric assay and was given as relative fluorescence units/Actin (RFU/Actin). Analyses were performed on protein lysates derived from *bcr-abl*-negative (**A**) control cells (NHDF, UROtsa) and from p210BCR-ABL-positive cells with the *bcr-abl* breakpoint variant b2a2 (**B**) (KCL-22, BV-173) and variant b3a2 (**C**) (LAMA-84, K562). Each data point corresponds to one single experiment. The protein levels of Separase, Securin, and CyclinB1 based on densitometric evaluation of immunostained Western blots were normalized to Actin as loading control. The corresponding Western blot images are cropped sections derived from stripped and reprobed Western blot immunostainings used for acquisition of densitometric data shown in Table 4. At least triplicate Western blot experiments were performed, one representative composite is depicted. Only significant p-values as calculated between treated and untreated cells were shown (see Table 4 for summarized Δ-values). Abbreviations: RFU, relative fluorescence units; IM, imatinib.

For non-malignant cells (NHDF, UROtsa) a tendency to dose-dependent decrease in Separase protein levels were observed in Western blot immunostaining experiments after IM exposure (Fig 2, Table 4). Protein levels dropped (range 30 to 34%) at IM concentrations of 5 μM. Separase proteolytic activity seems tightly linked to protein levels as dose-dependent decreases in proteolytic activity were found in the IM-treated cell lines (Fig 2A). Relative Separase activity losses of 1.2 (CI95%: -9.8 to 7.4) and 34% (CI95%: -65 to -3.1) were observed in NHDF and UROtsa cells at concentrations of 5 μM IM, respectively (Table 4). Separase activity downregulation concurs with increases in Securin and CyclinB1 protein levels, both posttranslational inhibitors of Separase.

**Table 4:** Percent changes (Δ-values are differences between means) in protein levels and proteolytic activity of Separase after IM treatment when compared to corresponding untreated cells.

Cell line, period of treatment	Separase protein levels [%] ^1^ ^,^ ^2^	Securin protein levels [%] ^1^ ^,^ ^2^	CyclinB1 protein levels [%] ^1^ ^,^ ^2^	Separase proteolytic activity [%]	Separase proteolytic activity [fold change] ^3^
**NHDF, 5 μM IM, 6d**	-34.4 (-54.0 to -14.9), p = 0.0032	+67.7 (-24.9 to 160.3), p = 0.1302	+59.4 (-88.8 to 207.6), p = 0.2920	-1.2 (-9.8 to 7.4), p = 0.7817	1.5 (1.2 to 2.0)
**UROtsa, 5 μM IM, 6d**	-30.0 (-83.8 to 23.9), p = 0.2124	+27.3 (-26.2 to 80.9), p = 0.2583	+36.4 (-177.2 to 250.0), p = 0.5399	-34.2 (-65.2 to -3.1), p = 0.0328	0.9 (0.6 to 2.1)
**KCL-22, 2.5 μM IM, 24h**	-11.8 (-98.7 to 75.1), p = 0.7249	-45.6 (-98.0 to 6.7), p = 0.0769	-44.6 (-69.6 to -19.7), p = 0.0047	+7.4 (-2.1 to 16.9), p = 0.1248	1.2 (0.7 to 4.4)
**BV-173, 2.5 μM IM, 48h**	-6.5 (-25.0 to 12.0), p = 0.4797	+9.7 (-32.4 to 51.7), p = 0.6101	+7.1 (-19.9 to 34.1), p = 0.5931	+2.5 (-24.4 to 29.5), p = 0.8457	1.1 (1.0 to 1.2)
**LAMA-84, 2.5 μM IM, 24h**	-75.8 (-119.1 to -32.5), p = 0.0064	-82.1 (-96.3 to -68.0), p<0.0001	-34.2 (-71.7 to 3.4), p = 0.0676	+31.1 (-0.04 to 62.2), p = 0.0502	5.4 (**)
**K562, 1 μM IM, 24h**	-56.9 (-77.4 to -36.5), p = 0.0015	-44.3 (9.5 to 98.2), p = 0.0844	-9.7 (-72.0 to 52.6), p = 0.7166	+9.1 (2.3 to 16.0), p = 0.0121	2.5 (1.9 to 3.7)

^1^ Δ-values were calculated from significant data sets diagrammed in Fig 1. All values are normalized to Actin.

^2^ for representative corresponding immunostained Western blots see Fig 1.

^3^ calculated quotient of Separase proteolytic activity [%] / Separase protein level [%]. Example (K526): (100+9.1)/(100–56.9) = 2.5

Abbreviations: d, days; h, hours; IM, imatinib; +, increase;-, decrease.

The confidence interval (95%CI) is given in parentheses.

** Since the CI of the denominator includes zero, it is not possible to compute the CI of this quotient.

Analogous experiments were performed with the p210BCR-ABL-positive cell lines KCL-22, BV-173 (b2a2, Fig 2B), LAMA-84 and K562 (b3a2, Fig 2C). While in the p210BCR-ABL b2a2 cell lines KCL-22 and BV-173 no significant changes in Separase protein levels and proteolytic activity were detected, the CML cell lines LAMA-84 and K562 displayed sensitivity to IM after 24h. Considerable decreases in Separase protein levels were achieved for LAMA-84 and K562 with low doses (1 μM) of IM (Fig 2C) pointing to the strong proliferative p210BCR-ABL-dependency of these cell lines as discussed by others. [46] Separase activation is an essential step for completing mitosis and entering the next cell cycle round. Here, relative Separase protein decreased by 75.8% (CI95%: -119.1 to -32.5) and 56.9 (CI95%: -77.4 to -36.5)% in LAMA-84 and K562 cells at concentrations of 2.5 and 1 μM IM, respectively (Table 4). Despite the observed decrease in Separase protein levels, increased levels of Separase proteolytic activity were measured (Fig 2C). Increases of 31.1% (CI95%: -0.04 to 62.2) and 9.1% (CI95%: 2.3 to 16.0) were observed for LAMA-84 and K562 cells, respectively (Table 4). Confirming a previous data set this concurs with conspicuous loss in the corresponding inhibitory protein levels of Securin and CyclinB1. [42] To exemplify, Securin protein levels dropped by 82.1% (CI95%: -96.3 to -68.0) and 44.3 (CI95%: 9.5 to 98.2) in LAMA-84 and K562 cells, respectively. As a result, about 25% of the residual Separase protein perform about 130% proteolytic activity in LAMA-84 cells (as calculated from Table 4) meaning an approximate 5.4-fold increase in Separase activity when compared to the respective untreated cells. The same holds true for K562 cells where a 2.5-fold activity increase in protease activity was estimated. Thus, the inhibitory effect of IM on Separase protein expression seems to be counterbalanced by the increase in Separase proteolytic activity. In fact, this proposed compensatory mechanism leads to a 31% increase in overall Separase proteolytic activity (Table 4) in LAMA-84 and 9% increase in K562 cells when compared to untreated cells. One might argue that the observed effect may be due to IM-related changes in the cell cycle, i.e. increased proportion of cells entering anaphase. However, FACS analysis of tested cells revealed no breakpoint-specific differences neither in G2/M cell proportion nor in the apoptotic cell fraction (<12%) that could clarify the observed phenomenon of IM-induced Separase activation (data not shown). Further potential caveats related to myeloblastic/lymphoblastic features of the analyzed CML cell lines (KCL-22: b2a2 and myeloblastic; BV-173: b2a2 and lymphoblastic; LAMA-84: b3a2 and myeloblastic; K562: b3a2 and lymphoblastic) could be ruled out since the observed IM-related changes in Separase proteolytic activity correlate only with the splice variant but not with their morphological features.

### Downregulation of *Espl1*/Separase by RNAi stimulates Separase proteolytic activity in b3a2 and b2a2 p210BCR-ABL-positive cells

**Fig 3:**
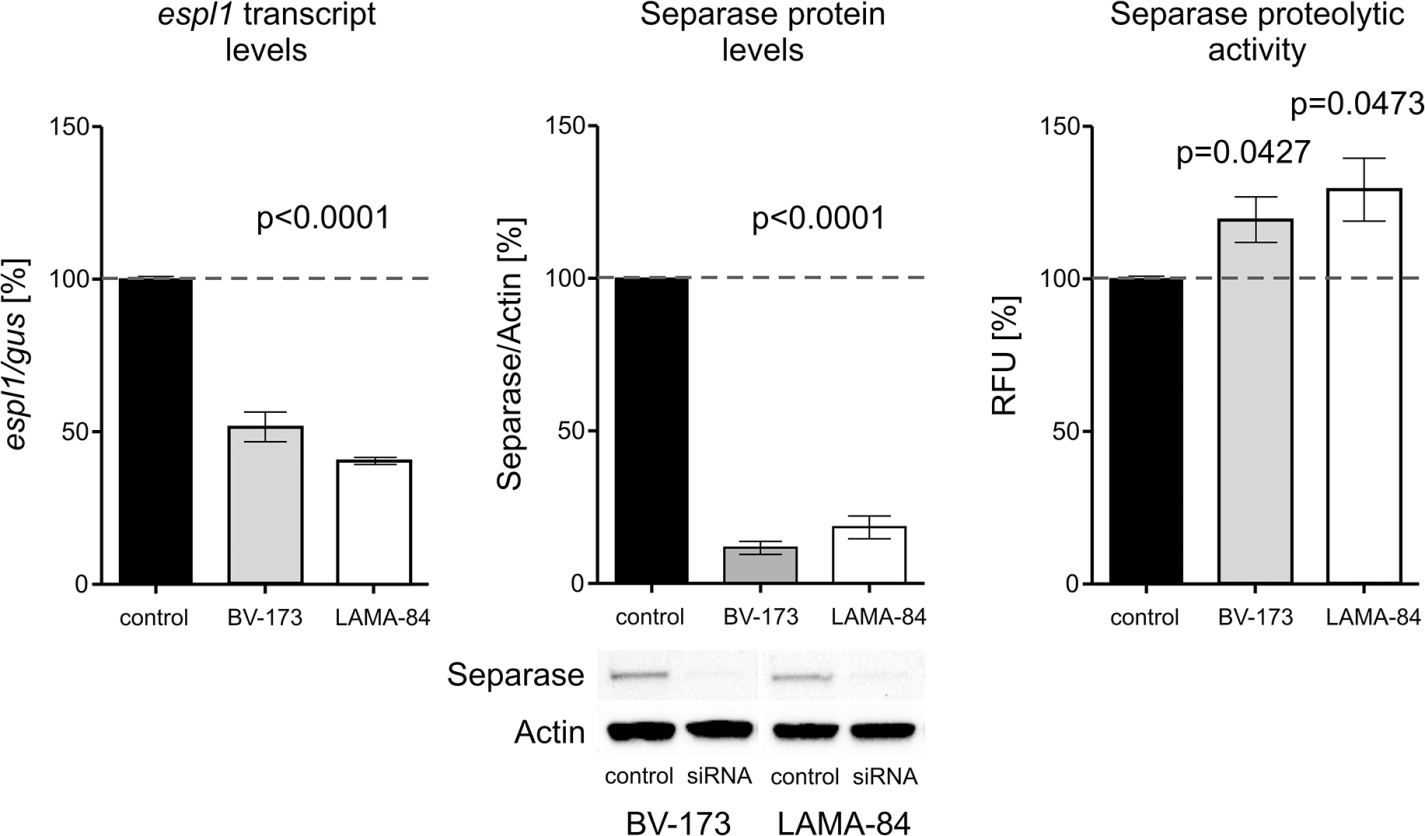
Separase expression and proteolytic activity after espl1 silencing by RNAi. BV-173 (b2a2) and LAMA-84 (b3a2) cells were treated with negative control siRNA (control) and espl1-specific siRNA (siRNA) for 48h. Consecutively, Separase expression was analysed on transcript level (qRT-PCR), protein level (Western blot immunostaining) and proteolytic activity level (fluorometric assay). All protein analyses were performed as described in legend to Fig 1. In qRT-PCR analysis the house-keeping gene gus (beta-glucuronidase) served as internal standard. Abbreviations: RFU, relative fluorescence units. As for interpretation, 11.7% of Separase protein (middle panel, p<0.0001) performs 118.5% proteolytic activity in BV-173 cells (right panel, p = 0.0427) corresponding to an about 10fold posttranslational activation of present Separase molecules (118.5%/11.7% = 10.1).

To test for the existence of a regulatory mechanism that may control Separase proteolytic activity in a Separase protein level-related manner, we silenced *espl1* transcript levels in BV-173 and LAMA-84 cells and monitored the influence of decreased transcript and protein levels on Separase proteolytic activity (Fig 3). Treatment of BV-173 and LAMA-84 cells with *espl1*-selective siRNA for 48h revealed decreased *espl1* transcript levels 53.8 (CI95%: -58.3 to -49.3) and 59.6 (CI95%: -66.2 to -52.9), respectively) and Separase protein levels 11.7 (CI95%: -15.1 to 38.5) and 18.5 (CI95%: -28.1 to 64.9), respectively). As a consequence, the Separase activity levels rose in both b2a2 and b3a2 cell lines reaching levels of 118.5% (CI95%: 95.5 to 141.6) (p = 0.0427) in BV-173 and 128.2 (CI95%: 85.4 to 171.1) (p = 0.0473) in LAMA-84 cells when compared to mock transfected control cells. Obviously, less Separase protein molecules performed more proteolytic activity resulting in an approximately 7-fold and 10-fold posttranslational activation of residual Separase molecules in LAMA-84 and BV-173 cells, respectively. This was mirrored by Securin and CyclinB1 protein levels (data not shown).

## Discussion

Our data show an about 4-fold (ratio 6.3 vs. 1.6) preference for acquisition of unbalanced over balanced ACA in IM-treated patients with the b3a2 fusion type when compared to patients with the b2a2 fusion. New unbalanced ACA under therapy had a negative impact on prognosis as they concurred with a higher incidence of BC in b3a2 patients (24% vs. 17%). These findings complement our earlier observations which demonstrated a negative prognostic impact of unbalanced ACA at diagnosis. [12] Moreover, it harmonises with previous data of the European LeukemiaNet stating that newly arising ACA under imatinib treatment and clonal evolution are considered features of acceleration and indicate failure of therapy. [3] This result points to existence of a molecular mechanism exclusively in b3a2 fusion type cells that merges chromosomal missegregation with imatinib treatment and an inhibited p210BCR-ABL tyrosine kinase. Several lines of evidence suggest also involvement of the centrosome:

i) Patel and Gordon reported that p210BCR-ABL and p145ABL are both centrosome-associated proteins that interact with the pericentriolar protein pericentrin. Furthermore, when CML cells were treated with imatinib there was a 55% and 20% reduction of p210BCR-ABL and p145ABL binding to pericentrin, respectively. They also observed abnormally high levels of Separase in CML CP and BC cells in comparison to normal CD34+ cells. [24] ii) We have previously demonstrated that supernumerary centrosomes are an early detectable feature in CML and contribute to chromosomal instability. In CML BC an increase in the percentage of centrosome aberrant cells significantly correlated with aneuploid karyotypes. [17] The expression of p210BCR-ABL (TetOn system) is able to drive centrosomal amplification and clonal evolution in a human *in vitro* CML model. [23] iii) Treatment of normal, p210BCR-ABL-negative and disease-unrelated cells with imatinib or other TKI induced centrosome aberrations *in vitro* and *in vivo*. [26,29]

Based on these cumulative data, we set out to investigate the role of Separase as a major key player in centrosomal duplication and chromosomal segregation during mitosis and to perform protein biochemical studies on Separase expression and activity profiles in human CML cells.

However, the study of Separase in clinical samples from CML patients under TKI treatment is associated with several problems. The major difficulty is the limited amount of *bcr-abl*-positive tumor cells under IM treatment. Under IM treatment about 70% of patients show fast response with *bcr-abl* transcript levels ≤10% at 3 months, ≤1% at 6 months and ≤0.1% (= MMR, major molecular response) from 12 months onward. Frequently, deeper molecular responses with a *bcr-abl* log reduction on the international scale (IS) of 4 (MR^4^) or 4.5 (MR^4.5^) are achieved. [49] Accessing routine blood samples it is impossible to isolate a sufficient number of *bcr-abl*-positive progenitor cells for experiments. Patients with AP and BC are very rare and bone marrow samples are hardly available. Moreover, individual co-morbidities and/or co-medications may influence the proliferation of target cells and hence weaken experimental results.

To overcome these difficulties, we conceived a systematic study using traditional cell culture systems representing a panel of thoroughly characterized CML cell lines. This approach offers a much more homogenous genetic background than clinical samples and provides the appropriate controls to evaluate Separase in the absence of IM or after RNAi silencing. We investigated the influence of IM on Separase protein levels and Separase proteolytic activity in a panel of six well characterized human cell lines with focus on cells expressing the b3a2 (K562, LAMA-84) and b2a2 (KCL-22, BV-173) fusion type of p210BCR-ABL. These experiments confirmed and expanded our recently published data where solely b3a2 cell lines have been investigated. [42]

We were able to confirm our recent data on K562 and LAMA-84 cell lines (b3a2) where IM treatment led to considerable decreases in Separase protein levels and unexpected activation of Separase proteolytic activity (Fig 2C). The approximately 5.4-fold and 2.5-fold (CI95%: 1.9 to 3.7) (Table 4) increase in Separase activity in LAMA-84 and K562 cells, respectively, confirms that Separase proteolytic activity is controlled on posttranslational level and independent of p210BCR-ABL tyrosine kinase activity. [42]

In contrast, in b2a2 fusion type cells (KCL-22, BV-173) and in non-malignant UROtsa cells we found slightly decreased or unchanged Separase protein levels. This becomes apparent in the calculated quotients of Separase activity [%]/Separase protein level [%] (Table 4). Quotients ranging around 1.0 (UROtsa, 0.9; KCL-22, 1.2; BV-173, 1.1) point to a constant activity/protein ratio. Slight alterations compared to corresponding untreated cells are probably due to the antiproliferative effects of IM in BCR-ABL-positive cells (KCL-22, BV-173) as IM treatment resulted in slight decreases in the proportion of G2/M and S phase cells while the amount of apoptotic cells was stable below 12% (data not shown). When we tried to mimic the observed posttranslational upregulation of Separase proteolytic activity in b3a2 cells by RNAi-related *espl1* silencing (Fig 3) we observed a striking increase in Separase activation in both b3a2 (LAMA-84, 7-fold) and b2a2 (BV-173, 10-fold) cells.

Overall, our results point to the existence of two corresponding mechanisms contributing to the observed Separase regulation in p210BCR-ABL positive cells. i) Selective downregulation of Separase protein levels by IM treatment exclusively in b3a2 cells, but not in b2a2 cells. ii) Posttranslational hyperactivation of the remaining Separase molecules to functionally compensate for the dropped Separase protein levels. This latter mechanism is p210BCR-ABL-dependent as it was not observed in NHDF cells but it does not rely on the kinase activity of p210BCR-ABL because hyperactivation occurred in face of IM treatment. Since hyperactivation of Separase proteolytic activity can be induced also in b2a2 fusion type cells (BV-173) by siRNA-mediated downregulation of Separase protein levels, the responsible actuator seems to be solely associated with the diverging response of b3a2 and b2a2 to IM treatment. Biochemically, it may be traced back to the additional 25 amino acid residues within the b3a2 p210BCR-ABL oncoprotein.

Studies from the pre-IM era have proposed that a sequential arrangement of six hydrophobic amino acid residues (F-L-N-V-I-V) encoded by *bcr* exon e14 (b3) may alter juxtaposition of p210BCR-ABL domains and subsequently, may change the binding kinetics for interacting protein partners. [50] Therefore, we speculate that differential protein interacting capabilities of the b3a2 and b2a2 fusion proteins may alter either expression or stability of Separase in IM treated CML cells. So far, the corresponding regulatory protein partners are unknown, but a varying recruitment of p210BCR-ABL to the centrosome may conceivable. [24]

The observed hyperactivation of Separase in b3a2 cells correlates with our cytogenetic data and points to higher “escape skills” of b3a2 leukemic stem cells that obviously outperform b2a2 cells in terms of unbalanced ACA acquisition and clonal evolution. The increased potential for clonal evolution and development of resistance may help b3a2 progenitors to escape the immunological and therapeutic pressure in terms of the Darwinian tumor model (“survival of the fittest”). [51] This assumption is concordant with the pathology of CML: Development of resistance in patients undergoing IM therapy frequently concurs with clonal evolution, which points to clonal evolution as a mechanism of resistance. [52,53] Furthermore, under IM the outcome of patients with clonal evolution is significantly inferior compared to those without. [6] It is therefore tempting to speculate that the IM-related hyperactivation of Separase proteolytic activity exclusively in b3a2 *bcr-abl*-positive cells is the best candidate mechanism for explaining promotion of tumor heterogeneity and clonal evolution. Even in dormant p210BCR-ABL low-expressing quiescent stem cells, this mechanism may eventually create descendant cell populations with enhanced fidelity to escape therapeutic pressure. [4,7] This suits the general opinion that newly arising ACA under treatment are a clear sign of therapy failure. [3,12] Our data seem to contradict the general opinion that b3a2 patients show better molecular responses than b2a2 patients. However, no difference in cytogenetic response and overall survival has been previously reported when comparing the impact of b3a2 and b2a2 splice variants on disease phenotype and outcome under imatinib therapy. [54] Therefore, we conclude that our proposed Separase-related mechanism favoring specifically the acquisition of unbalanced ACA in b3a2 patients has no detectable influence on clinical response and overall survival. However, our clinical results clearly demonstrate that there are differences between b2a2 and b3a2 patients during IM treatment in terms of ACA type (unbalanced preferred) and time to blast crisis. These differences seem to be clinically relevant. Because of the rather small sample size and the relatively low number of statistical tests performed (4) we refrained from Bonferroni correction in order to control the overall type I error rate. Otherwise, differences between both groups may have been missed. However, even if the p value is multiplicated by 4 (according to Bonferroni correction) the difference regarding “time to BC” remains statistically significant (p = 0.0496) whereas the difference regarding the p210BCR-ABL splice variant shows a trend (p = 0.0984).

In conclusion, our data indicate the existence of a p210BCR-ABL-related feedback mechanism that posttranslationally stimulates Separase proteolytic activity after an IM-induced decrease in Separase protein levels exclusively in b3a2 fusion type CML cells. As a consequence, b3a2 cells may be more susceptible to chromosomal missegregation (aneuploidy) and clonal evolution than leukemic b2a2 progenitors. Prospective studies on the Separase regulatory network in CML may give rise to new concepts in carcinogenesis and leukemia therapy using selective Separase inhibitors. [55]

## Supporting Information

S1 Table Cytogenetic data of CML patients with ACA in the Ph+ clone acquired during IM treatment. (DOC)
