# Supplementary material for: Clonal Evolution and Blast Crisis Correlate with Enhanced Proteolytic Activity of Separase in BCR-ABL b3a2 Fusion Type CML under Imatinib Therapy
Source: PLoS One. 2015 Jun 18;10(6):e0129648. doi: 10.1371/journal.pone.0129648 (PMC4472749; doi:10.1371/journal.pone.0129648)
Supplement: S1 Table — (DOC) [file pone.0129648.s001.doc]

**S1 - Table. Cytogenetic data of CML patients with ACA in the Ph+ clone acquired during IM treatment.**

| **No.** | **Sex** | **breakpoint variant** | **Type of ACA** | **Blast crisis *** | **Time to BC in months** | **ACA karyotype within the Ph+ clone** |
| --- | --- | --- | --- | --- | --- | --- |
| 1 | f | b2a2 | balanced | no |  | 46,XX,t(2;16)(p2?3;p1?3),t(9;22)(q34;q11)[26] |
| 2 | m | b2a2 | balanced | no |  | 46,XY,inv(7)(p22q32),t(9;22)(q34;q11)[25] |
| 3 | f | b2a2 | balanced | no |  | 46,XX,t(1;21)(q21;q22),t(9;22)(q34;q11)[7] |
| 4 | m | b2a2 | balanced | no |  | 46,XY,t(4;6)(q21;p23),t(9;22)(q34;q11)[20] |
| 5 | m | b2a2 | balanced | no |  | 46,XY,t(9;22)(q34;q11),t(14;17)(p11;p11) |
| 6 | m | b2a2 | balanced | no |  | 46,XY,t(9;22)(q34;q11),t(15;20)(q13;p12)[20] |
| 7 | m | b2a2 | balanced | yes | 27 | 46,XY,t(3;8)(q2?6;q24),t(9;22;7)(q34;q11.2;q11.2) |
| 8 | m | b2a2 | balanced | no | 48 | 46,XY,inv(3)(q21q26),t(9;22)(q34;q11)[9] |
| 9 | m | b2a2 | balanced | no |  | 46,XY,t(2;12)(q33;p13),t(9;22)(q34;q11)[20] |
| 10 | m | b2a2 | balanced | no |  | 46,XY,t(1;12)(p24;q24),t(1;9;22)(p36;q34;q11) |
| 11 | m | b2a2 | balanced | yes | 24 | 46,XY,t(3;11)(q26;q23),t(9;22)(q34;q11)[20] |
| 12 | m | b2a2 | balanced | no |  | 46,XY,t(3;21)(q26;q22),t(9;22)(q34;q11)[16] |
| 13 | m | b2a2 | balanced | no |  | 46,XY,t(6;15),t(9;22)(q34;q11)[6] |
| 14 | m | b2a2 | balanced | no |  | 46,XY,inv(3)(q21q26),t(9;22)(q34;q11)[24] |
| 15 | m | b2a2 | unbalanced | yes | 35 | 46,XY,t(9;22)(q34;q11)[18]/47,idem,+der(22)t(9;22)(q34;q11)[2] |
| 16 | f | b2a2 | unbalanced ** | yes | 18 | 47,XX,+8,t(9;22)(q34;q11),i(17)(q10) |
| 17 | m | b2a2 | unbalanced | yes | 14 | 46,XY,t(9;22)(q34;q11)[16]/50,idem,+8,+12,+18,+21[3] |
| 18 | m | b2a2 | unbalanced | no |  | 47,XY,+8,t(9;22)(q34;q11)[16] |
| 19 | m | b2a2 | unbalanced | no |  | 46,XY,t(9;22)(q34;q11)/45,XY,t(9;22)(q34;q11),-21[4] |
| 20 | f | b2a2 | unbalanced | no |  | 47,XX,+8,t(9;22)((q34;q11)[10] |
| 21 | f | b2a2 | unbalanced | no |  | 46,XX,t(9;15;22)(q34;q26;q11)[22]/48,idem,+8,+19[3] |
| 22 | m | b2a2 | unbalanced | no |  | 47,XY,t(9;22)(q34;q11)[18]/47,idem,+8[6] |
| 23 | f | b2a2 | unbalanced | no |  | 46,XX,der(9)t(9;22)(q34;q11),ider(22)(q10)[13] |
| 24 | m | b2a2 | unbalanced | no |  | 47,XY,+8,t(9;22)(q34;q11)[1]/48,idem,+der(22)t(9;22)(q34;q11)[1]/46,XY[21] |
| 25 | f | b2a2 | unbalanced | no |  | 46,XX,t(9;22)(q34;q11.2),add(20)(p11.2)[9] |
| 26 | f | b2a2 | unbalanced | no |  | 46,XX,del(1)(q32),der(9)t(1;9)(q32;q34)t(1;22)(q44;q11),der(22)t(9;22)(q34;q11) |
| 27 | m | b2a2 | unbalanced | no |  | 48,XY,+8,t(9;22)(q34;q11),+der(22)(t(9;22)(q34;q11)[18] |
| 28 | m | b2a2 | unbalanced | no |  | 46,XY,t(9;22)(q34;q11),i(9)(p10),der(17)t(9;17) |
| 29 | f | b2a2 | unbalanced | no |  | 46,XX,del(1)(q21),der(9)t(9;22)(q34;q11),der(22)t(9;22)(q34;q11)t(1;9)(q21;q34)[20] |
| 30 | f | b2a2 | unbalanced | no |  | 47,XX,+8,t(9;22)(q34;q11)[4] |
| 31 | m | b2a2 | unbalanced | yes | 6 | 47,XY,+8,t(9;22)(q34;q11)[17];49,XY,+8,t(9;22)(q34;q11),+19,+20[7] |
| 32 | m | b2a2 | unbalanced | no |  | 46,XY,del(5)(q11q14),t(9;22)(q34;q11)[19] |
| 33 | m | b2a2 | unbalanced | no |  | 49,XY,+8,t(9;22)(q34;q11),+10,+der(22)[13] |
| 34 | f | b2a2 | unbalanced | no |  | 46,XX,del(X)(p?21),t(9;22)(q34;q11)[6] |
| 35 | m | b2a2 | unbalanced | no |  | 47,XY,+8,t(9;22)(q34;q11)[4] |
| 36 | m | b2a2 | unbalanced | yes | 26 | 46,XY,der(7)r(7)(p11q32)del(7)(q11q22),del(9)(p12p24),t(9;22)(q34;q11) |
| 1 | f | b3a2 | balanced | yes | 5 | 46,XX,t(3;21)(q26;q22),t(9;22)(q34;q11)[15] |
| 2 | m | b3a2 | balanced ** | no |  | 46,XY,der(6)t(6;17)(p21;q11),t(9;22)(q34;q11)[11] |
| 3 | m | b3a2 | balanced | yes | 14 | 46,XY,t(9;22)(q34;q11),inv(16)(p13q22)[4] |
| 4 | f | b3a2 | balanced | no |  | 46,XX,t(9;22)(q34;q11.2)[7]/46,XX,t(9;22)(q34;q11.2),der(19)t(19;?)(p13.3;?)[13] |
| 5 | f | b3a2 | unbalanced | no |  | 46,XX,der(2)t(2;4)(q37;q21),del(4)(q21),t(9;22)(q34;q11) |
| 6 | m | b3a2 | unbalanced | yes | 9 | 44,XY,der(9)t(9;22)(q34;q11)add(9)(q34),-11,-12,dic(16;?)(p13;?)[20] |
| 7 | m | b3a2 | unbalanced ** | yes |  | 48,XY,+8,t(9;22)(q34;q11),i(17)(q10),+der(22)t(9;22)(q34;q11)[10] |
| 8 | m | b3a2 | unbalanced ** | yes | 3 | 50,XY,+8,+8,t(9;22)(q34;q11),+14,i(17)(q10),+der(22)t(9;22)(q34;q11) |
| 9 | m | b3a2 | unbalanced | no |  | 46,XYt(9;22)(q34;q11)[14]/46,XY,der(9)t(9;22)(q34;q11),ider(22)(q10)t(9;22)(q34;q11)[11] |
| 10 | f | b3a2 | unbalanced | no |  | 46,XX,del(3)(p11p21),t(9,22)(q34;q11)[13] |
| 11 | m | b3a2 | unbalanced | no |  | 47,XY,t(9;22)(q34;q11),+der(22)t(9;22)(q34;q11)[14] |
| 12 | m | b3a2 | unbalanced | Yes | 15 | 48,XY,+8,t(9;22)(q34;q11),+19[29] |
| 13 | m | b3a2 | unbalanced | no |  | 47,XY,t(9;22)(q34;q11),+der(22)t(9;22)(q34;q11)[11] |
| 14 | m | b3a2 | unbalanced | yes | 9 | 46,XY,t(9;22)(q34;q11)[14]/47,XY,t(9;22)(q34;q11),+der(22)t(9;22)(q34;q11)[6] |
| 15 | m | b3a2 | unbalanced | no |  | 46,XY,t(9;22)(q34;q11)[7]/47,XY,+8,t(9,22)(q34;q11)[2]/48,idem,+der(22)t(9;22)(q34;q11)[3] |
| 16 | m | b3a2 | unbalanced | no |  | 46,XY,t(9;22)(q34;q11)[17]46,XY,dup(1)(q31q21),t(9;22)(q34;q11)[3] |
| 17 | m | b3a2 | unbalanced | no |  | 46,XY,t(9 ;22)(q34;q11)[23]/47,XY,t(9;22)(q34;q11),+der(22)t(9;22)(q34;q11)[2] |
| 18 | m | b3a2 | unbalanced | no |  | 48,XY,t(9;22)(q34;q11),+19,+der(22)t(9;22)(q34;q11)[2]/45,XY,-7[9] |
| 19 | f | b3a2 | unbalanced | no |  | 47,XX,t(3;9;22)(q?;q34;q11),+der(7)del(7)(p11)del(7)(q11) |
| 20 | m | b3a2 | unbalanced | no |  | 47,XY,+8,t(9;22)(q34;q11)[19] |
| 21 | f | b3a2 | unbalanced | yes | 9 | 45,XX,der(7;9)(q10;q10),t(9;22)(q34;q11) |
| 22 | m | b3a2 | unbalanced | no |  | 47,XY,t(9;22)(q34;q11),+der(22)t(9;22)(q34;q11)[4] |
| 23 | m | b3a2 | unbalanced | no |  | 45,XY,inv(3)(q21q26),-7,t(9;22)(q34;q11)[11] |
| 24 | f | b3a2 | unbalanced | no |  | 47,XX,+8,t(9;22)(q34;q11)[3] |
| 25 | m | b3a2 | unbalanced | no |  | 47,XY,+8,t(9;22)(q34;q11)[4] |
| 26 | m | b3a2 | unbalanced | no |  | 45,X,-Y[6]/46,XY,t(9,22)(q34;q11),add(8)(q24)[2]/45,X,-Y,der(8)t(10;18)(q11;p11)[10] |
| 27 | m | b3a2 | unbalanced | no |  | 48,XY,+8,t(9;22)(q34;q11),+der(22)t(9;22)(q34;q11)[20] |
| 28 | m | b3a2 | unbalanced | no |  | 47,XY,+8,t(9;22)(q34;q11),idic(17)(p11)[24] |
| 29 | f | b3a2 | unbalanced | no |  | 46,XX,der(7;11)ins(7;11)(p14;p11q25)t(7;11)(p22;q?) |

ACA, additional clonal cytogenetic alteration; IM, imatinib; BC, blast crisis

* within mean observation time of 5.6 years

** TP53 deletion
